# Trauma Registry: Trauma Quality indicators analysis in hospitalized patients

**DOI:** 10.1590/0100-6991e-20243604-en

**Published:** 2024-03-20

**Authors:** LUCA GIOVANNI ANTONIO PIVETTA, PEDRO DE SOUZA LUCARELLI ANTUNES, GIOVANNA MENNITTI SHIMODA, JOSÉ GUSTAVO PARREIRA, JACQUELINE ARANTES GIANNINNI PERLINGEIRO, JOSE CESAR ASSEF

**Affiliations:** 1 - Irmandade da Santa Casa de Misericórdia de São Paulo, Serviço de Emergência - São Paulo - SP - Brasil; 2 - Irmandade da Santa Casa de Misericórdia de São Paulo, Departamento de Cirurgia - São Paulo - SP - Brasil; 3 - Faculdade de Ciências Médicas da Santa Casa de São Paulo - São Paulo - SP - Brasil

**Keywords:** Traumatology, Trauma Severity Indices, Quality of Health Care, Traumatologia, Índices de Gravidade do Trauma, Qualidade da Assistência à Saúde

## Abstract

**Purpose:**

to consolidate a Trauma Register (TR) trough REDCap data acquisition platform and to validate, in this context, local Quality Indicators (QI) as improvement opportunities in trauma management.

**Methods:**

continuous data acquisition of all patients admitted in Irmandade da Santa Casa de Misericórdia de São Paulo adult Trauma bay and it’s validation in REDCap platform; 6 months retrospective cohort of QI impact in length of hospitalar stay, complications and mortality. Fisher, Chi-squared, Wilcoxon and Kruskal-Wallis tests were used to correlate QIs fails with the endpoints, considering p<0.05 and CI <95% as statically significant.

**Results:**

465 were admitted in Trauma bay, with 137 patients hospitalized (29.5%); the number of QIs compromised were related with more complications (p=0.075) and increased length of stay (p=0.028), especially the delay in open fracture’s surgical management, which increased the severe complications’ incidence (p=0.005).

**Conclusion:**

the REDCap data acquisition platform is useful as a tool for multi center TR implementation, from ethical and logistical point of view; nevertheless, the proposed QIs are validated as attention points in trauma management, allowing improvements in traumatized patients treatment.

## RESUMO

**Objetivo:**

implementação de Registro de Trauma (RT) através da plataforma REDCap e validação dos Filtros de Qualidade (FQ) desenvolvidos como pontos de melhora no atendimento ao traumatizado.

**Métodos:**

implementação de coleta contínua dos dados de todos os pacientes adultos admitidos na sala de Trauma da Irmandade da Santa Casa de Misericórdia de São Paulo e validação destes na plataforma REDCap; realizada coorte retrospectiva dos dados validados em um período de 6 meses e o impacto dos FQ desenvolvidos no tempo de internação hospitalar, complicações e mortalidade dos pacientes. A correlação do comprometimento dos FQ com os desfechos foi obtida através dos testes de Fisher, Qui-Quadrado, Wilcoxon e Kruskal-Wallis, considerando p<0,05 e IC 95% como significativos.

**Resultados:**

incluídos no estudo 465 pacientes admitidos no período de estudo, com necessidade de internação em 137 casos (29,5%); o número de FQs comprometidos relacionou-se com maior número de complicações (p=0,075) e maior tempo de internação (p=0,028), sobretudo o atraso na ida ao Centro Cirúrgico de fraturas expostas, que aumentou a incidência de complicações graves (p=0,005).

**Conclusão:**

a plataforma REDCap é adequada e útil na implementação de RT, permitindo o uso ético e multicêntrico de dados; os FQs propostos determinam pontos de atenção a serem revistos no atendimento do trauma, permitindo melhorias na qualidade de atendimento ao paciente traumatizado.

## INTRODUCTION

Trauma is an important cause of medical care in all areas of society. Death from external causes is the most important when considering the age group of productive adults, surpassing all other potentially fatal diseases^1^ ^-^ ^4^. According to DATASUS, in 2020 there were 146,038 deaths in Brazil due to external causes, of which 47,647 were assaults (ICD10 X91-Y09), 81,363 due to transportation accidents, (ICD10 V01-V99), and 15,742 due to falls, 8,734 being falls from the same level^5^ ^,^ ^6^.

The first intervention in the bimodal curve of mortality resulting from traumatic injuries occurs with prevention^7^ ^,^ ^8^. If this fails, the quality of hospital care for traumatized patients is a crucial point. This means that the impact of trauma on health systems is considerable. The cost of deaths and injuries secondary to traffic accidents can reach 2% of the gross domestic product (GDP) of countries with high per capita income and up to 5% of GDP in countries with low per capita income^7^.

To optimize resources and improve care for traumatized patients, among many mechanisms^9^, Quality Programs, through review of medical records, morbidity and mortality meetings, study of preventable deaths, monitoring of audit filters, establishment of review committees of morbidity and mortality, closing the cycle with the team, and, mainly the implementation of Trauma Registries^10^ ^-^ ^14^ become clinical-epidemiological tools for tracking points of failure in care. Such quality programs are essential for structured trauma care^9^.

International studies indicate a drop in mortality in places where the quality model based on the Trauma Registry is implemented^19^ ^,^ ^20^. In developed countries, the health system is based on information collected in large databases, in a continuous process of cooperation and coordination of actions with the aim of improving medical care^12^. In the United States of America, the American College of Surgeons developed the National Trauma Data Bank^®^ (NTDB), which in 2006 already accumulated around two million cases collected, with information from 640 trauma centers in 45 American states^2^. In Canada, the discussion of the need to implement a national trauma registry deepened in 1997. The Canadian Institute for Health Information (CIHI) began collecting data in the National Trauma Registry (NTR) in 2001^3^.

The use of trauma severity indices (anatomical and physiological^15^ ^-^ ^18^) and the monitoring of audit filters (AF) helps to identify possible preventable or potentially avoidable deaths, aiding in recognizing points for improvement in care^19^. The cycle closes when we collect the data again, to evaluate the response to the established plan.

[Table t1]

**Table 1 t1:** Quality Filters (QF) used in the analysis of care for the studied population.

QF1 Unidentified injury within 24 hours
QF2 Time until drainage of acute subdural hematoma >4h
QF3 Chest re-drainage
QF4 Open fracture referral to the Operating Room >6h
QF5 Exploratory Laparotomy for hypotensive patients >60 min
QF6 Failure to activate Massive Transfusion Protocol (ABC >2 or Shock Index > .2)
QF7 Lack of Respiratory Frequency record upon admission
QF8 Change in vital signs without analysis of arterial blood gases and lactate
QF9 Absence of Pre-hospital Vital Signs recorded in the medical chart
QF10 Computerized Tomography report delivered in >6h

“Audit filters” or Quality Filters (QF) are variables proposed by the assistants of each service to be monitored and which, when present, can represent opportunities for improving the system^20^ ^-^ ^25^. An additional definition understands that QF would be “sentinel events” that could be related to a worse prognosis or inadequate treatment. Once these events are identified, this would trigger the Quality Program (QP) peer review of the case and, should any failure be identified, measures to prevent future events would be implemented. The Trauma Committee of the American College of Surgeons, in 1990, proposed 22 AFs that were initially used by the various quality programs^26^. However, the benefit of an AF must be considered locally, adapting to the local realities and demands of each service, as proposed by Stewart et al. in 2016^27^. They must also be audited, according to the study by Horton et al. in 2017^28^.

In accordance with this need to adapt studies to local realities, the filters selected in the work of Wu et al.^29^, developed for the reality of Cameroon, are not identical to those used by Berg et al.^30^, in India, or those evaluated in the study by Bieler et al.^27^ and Zhang, GX et al.^10^.

In Brazil, some institutions promote data collection for specific studies, but without national unification, despite the recognition that a Trauma Registry allows monitoring results and identifying some areas that need to be improved^24^ ^-^ ^26^. At the Irmandade da Santa Casa de Misericórdia de São Paulo (ISCMSP), since 2013, data on trauma patients has been collected as part of the Traumatized Care Quality Program (PQAT).

The primary objective of this study is the implementation of a continuous Trauma Registry in the ISCMSP Emergency Service, with potential multicenter use, through the REDCap data acquisition platform. As a secondary objective, we have the validation of the QF developed at ISCMSP as points of improvement in trauma care, enabling use by other services and agencies, with adaptation to their reality.

## METHODS

Trauma is an important cause of medical care in all areas of society. Death from external causes is the most important when considering the age group of productive adults, surpassing all other potentially fatal diseases^1^ ^-^ ^4^. According to DATASUS, in 2020 there were 146,038 deaths in Brazil due to external causes, of which 47,647 were assaults (ICD10 X91-Y09), 81,363 due to transportation accidents, (ICD10 V01-V99), and 15,742 due to falls, 8,734 being falls from the same level^5^ ^,^ ^6^.

The first intervention in the bimodal curve of mortality resulting from traumatic injuries occurs with prevention^7^ ^,^ ^8^. If this fails, the quality of hospital care for traumatized patients is a crucial point. This means that the impact of trauma on health systems is considerable. The cost of deaths and injuries secondary to traffic accidents can reach 2% of the gross domestic product (GDP) of countries with high per capita income and up to 5% of GDP in countries with low per capita income^7^.

To optimize resources and improve care for traumatized patients, among many mechanisms^9^, Quality Programs, through review of medical records, morbidity and mortality meetings, study of preventable deaths, monitoring of audit filters, establishment of review committees of morbidity and mortality, closing the cycle with the team, and, mainly the implementation of Trauma Registries^10^ ^-^ ^14^ become clinical-epidemiological tools for tracking points of failure in care. Such quality programs are essential for structured trauma care^9^.

International studies indicate a drop in mortality in places where the quality model based on the Trauma Registry is implemented^19^ ^,^ ^20^. In developed countries, the health system is based on information collected in large databases, in a continuous process of cooperation and coordination of actions with the aim of improving medical care^12^. In the United States of America, the American College of Surgeons developed the National Trauma Data Bank^®^ (NTDB), which in 2006 already accumulated around two million cases collected, with information from 640 trauma centers in 45 American states^2^. In Canada, the discussion of the need to implement a national trauma registry deepened in 1997. The Canadian Institute for Health Information (CIHI) began collecting data in the National Trauma Registry (NTR) in 2001^3^.

The use of trauma severity indices (anatomical and physiological^15^ ^-^ ^18^) and the monitoring of audit filters (AF) helps to identify possible preventable or potentially avoidable deaths, aiding in recognizing points for improvement in care^19^. The cycle closes when we collect the data again, to evaluate the response to the established plan.

“Audit filters” or Quality Filters (QF) are variables proposed by the assistants of each service to be monitored and which, when present, can represent opportunities for improving the system^20^ ^-^ ^25^. An additional definition understands that QF would be “sentinel events” that could be related to a worse prognosis or inadequate treatment. Once these events are identified, this would trigger the Quality Program (QP) peer review of the case and, should any failure be identified, measures to prevent future events would be implemented. The Trauma Committee of the American College of Surgeons, in 1990, proposed 22 AFs that were initially used by the various quality programs^26^. However, the benefit of an AF must be considered locally, adapting to the local realities and demands of each service, as proposed by Stewart et al. in 2016^27^. They must also be audited, according to the study by Horton et al. in 2017^28^.

In accordance with this need to adapt studies to local realities, the filters selected in the work of Wu et al.^29^, developed for the reality of Cameroon, are not identical to those used by Berg et al.^30^, in India, or those evaluated in the study by Bieler et al.^27^ and Zhang, GX et al.^10^.

In Brazil, some institutions promote data collection for specific studies, but without national unification, despite the recognition that a Trauma Registry allows monitoring results and identifying some areas that need to be improved^24^ ^-^ ^26^. At the Irmandade da Santa Casa de Misericórdia de São Paulo (ISCMSP), since 2013, data on trauma patients has been collected as part of the Traumatized Care Quality Program (PQAT).

The primary objective of this study is the implementation of a continuous Trauma Registry in the ISCMSP Emergency Service, with potential multicenter use, through the REDCap data acquisition platform. As a secondary objective, we have the validation of the QF developed at ISCMSP as points of improvement in trauma care, enabling use by other services and agencies, with adaptation to their reality.

## RESULTS

We included 465 trauma patients admitted during the study period in the analysis, aged between 14 and 102 years (average 41.4), the majority (79.8% - 371) being male and victims of minor trauma - Injury Severity Score less than 16 in 94.2% of cases (437/465).

Regarding the trauma mechanism, closed trauma was more common than penetrating one, fall from height being the most frequent mechanism (25.2%). Furthermore, most patients admitted were stable from a hemodynamic point of view, when considering Systolic Blood Pressure (average 129), Heart Rate (average 96.7), and Respiratory Rate (average 18.2) as parameters of instability, with only 7 cases of patients admitted in shock (1.5%) - Figure 1.

**Graphic 1:**
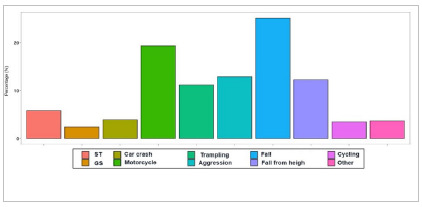
Trauma mechanism in patient sample (ST - Stabbing; GS - Gunshot).

There was a small proportion of patients studied who required interventions while still in the Trauma room, with 15 (3.2%) intubations, 14 (3%) chest drainages, and four (0.9%) activations of the Massive Transfusion Protocol.

The most affected segments, according to the Abbreviated Injury Score, were the extremities (109 - 23.4%) and skull (104 - 22.4%), followed by the face (61 - 13.3%), abdomen/pelvis (41 - 8.8%), and chest (36 - 7.7%). Despite being more common, though, traumas affecting the cranial segment were more serious than those affecting the extremities.

There were 17 skull bone fractures (3.65%), 21 facial fractures (4.7%), nine Epidural Hematomas (2%), eight Subdural Hematomas (1.8%), and 12 brain contusions (2.7%), 42 fractures of the upper limbs (9.1%), 50 of the lower limbs (10.8%) - with only one peripheral vascular injury -, 15 of the pelvis (3.2%), and 18 of the spine (3.9%). The frequency of potentially life-threatening injuries was small, with three (0.6%) airway injuries and two (0.4%) tension pneumothoraces.

Regarding intrabdominal, we found 11 liver injuries (2.2%), nine splenic lesions (1.7%), seven kidney injuries (1.3%), in addition to other less frequently affected organs (Figure 2). In 10 (2.2%) cases, non-operative treatment of parenchymal viscera injuries was instituted, with the aid of an Endovascular approach in three of them, with no failures noted.

**Graphic 2:**
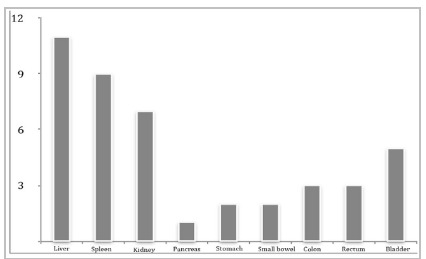
Intra-abdominal organs injured.

Regarding the need for surgical intervention, the rate of craniotomy was 0.6% (three cases), thoracotomy and videothoracoscopy 0.4% (two cases) each, videolaparoscopy 0.6% (three cases), laparotomy 3.9% (18 cases), and orthopedic approach 12.9% (60 cases). There were also seven cases (1.5%) of chest drainage in the operating room and one case in which Damage Control surgery was used, with laparostomy.

Regarding morbidity and mortality, 43 (9.2%) patients had some treatment deviation. We used The Clavien-Dindo Classification to stratify complications in these patients, of whom 16 fell into grades III and IV, requiring surgical intervention or presenting some organic dysfunction. There was one intra-abdominal infection, six surgical re-approaches (1.3%), eight patients developed some degree of renal dysfunction (1.7%), six of them (1.3%) required renal replacement therapy, and ten cases resulted in death (2.15%). The dropout rate due to treatment withdrawal was 14.6% (68), with the remaining 388 patients being discharged with clinical improvement.

Of all patients admitted to the Trauma room, 137 (29.5%) required hospital admission, a group investigated with greater attention. When analyzed regarding length of stay, the majority had a short period of hospitalization, less than seven days (51.1%), followed by those with seven to 30 days of hospitalization (40%), and those with prolonged stay (greater than 30 days), 8.9%.

As for trauma mechanisms, there was a greater number of patients who were victims of car accidents and penetrating injuries in the sample of hospitalized patients (Figure 3). Consequently, there was an increase in the need for intervention in the Trauma Room for these patients, such as orotracheal intubation and chest drainage, which increased from 3.2% and 3%, respectively, to 10.2%, and activation of the Massive Transfusion protocol, which tripled. This data is in line with the analysis of the severity of trauma patients, according to the AIS, which more than tripled in the population of hospitalized patients (19.1%).

**Graphic 3:**
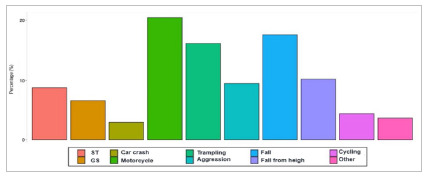
Trauma mechanism in patient who regard hospital admission (ST - Stabbing; GS - Gunshot).

In this sub-population, the Quality Filters proposed for studying trauma patient care were applied and related to morbidity and mortality. Each Quality Filter was analyzed considering the relevant subpopulation within the subgroup of hospitalized patients, as a way of eliminating potential selection or detection biases, with the number of compromised cases in relation to the total number of cases evaluated by each filter as set out below: FQ1 3/136 - 2.2%; FQ2 0; FQ3 4/25 - 16%; FQ4 15/29 - 51.7%; FQ5 2/6 - 33.3%; FQ6 1/136 - 0.7%; FQ7 20/116 - 14.7%; FQ8 1/136 - 0.7%; FQ9 127/136 - 93.4%; FQ10 41/136 - 30.1%.

In addition to the discriminative analysis of each compromised QF, we created a variable, hereinafter referred to as “QF Number”, to establish the severity of failures in trauma care, considering that the greater the compromise of points of care in the care chain, the greater the risk brought to the patient.

When analyzing the relationship between the impairment of QFs and morbidity and mortality parameters, we noted that due to the small sample of patients, it was not possible to establish a relevant statistical correlation between the Filters that refer to the presence of an unnoticed injury in the first 24 hours (FQ1) and Subdural Hematoma drainage time >4h (FQ2) and length of stay, complications, or mortality. Furthermore, we observed that chest re-drainage (FQ3), in addition to not being related to morbidity and mortality outcomes, was not related to trauma severity (p=0.604), not even in thoracic follow-up (p=0.812).

Regarding the brevity of the surgical approach to open fractures analyzed in FQ4 (Open fracture with referral to OR >6h), we observed a delay in the surgical approach related to the mechanism of trauma, more common in multiple traumas involving motorcyclists and pedestrian accidents (p=0.008) - Table 2.

**Table 2:** Frequency of the variables Trauma Mechanism and FQ4 (Open fracture with referral to the OR >6h) in the sample (p=0.008, Fisher’s Exact Test).

	No	Yes	Total
Gunshot Wound	2 (100%)	0 (0%)	2 (6.9%)
Motorcyclie accident	3 (27.3%)	8 (72.7%)	11 (37.9%)
Run over	1 (14.3%)	6 (85.7%)	7 (24.1%)
Assault	1 (100%)	0 (0%)	1 (3.4%)
Fall from standing height	4 (100%)	0 (0%)	4 (13.8%)
Fall from height	3 (75%)	1 (25%)	4 (13.8%)
Total	14 (48.3)	15 (51.7%)	29 (100%)

The delay in going to the OR had an impact, within the population of hospitalized patients, on the incidence of serious complications (p=0.005), requiring surgical intervention or generating organic dysfunctions, despite not having an impact on the length of stay (p=0.665) or on mortality (p=0.100).

On the other hand, the referral of patients admitted in shock to Exploratory Laparotomy in the first hour (FQ5) did not present a significant failure (two cases out of 465 - 0.4%). However, compromising the brevity of Laparotomy was associated with the occurrence of complications, especially more serious ones, including death (p=0.03).

Filters on non-activation of the Massive Transfusion Protocol when necessary (FQ6), absence of Respiratory Frequency recording on admission (FQ7), change in vital signs without analysis of arterial blood gases and lactate (FQ8), and absence of pre-hospital Vital Signs record in the medical chart (FQ9) were not related to length of stay, incidence of complications, or mortality of the traumatized patients. CT Report delivered in > 6h (FQ10) was also not related to the parameters of morbidity and mortality or length of stay (p=0.696).

The QF Number, which represents the number of patients with impairment of one to four QFs, could quantify the failure in trauma patient care. There was an established relationship of greater failure in care, represented by the greater number of compromised Quality Filters, with penetrating trauma mechanisms (p<0.001) - Figure 4.

**Graph 4:**
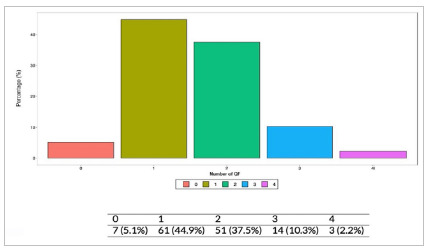
Frequency of Quality Filter number compromise at traumatized in-patient.

There was also an association between the severity of the extremity trauma, represented by higher AIS values, with the number of QFs affected (p=0.011), given that this is in line with the break in the chain of care observed when exposed fractures go to the OR. When severity was stratified with NISS, we observed a greater impairment of QFs in more severe traumas (p=0.03) - Figure 5.

**Graphic 5:**
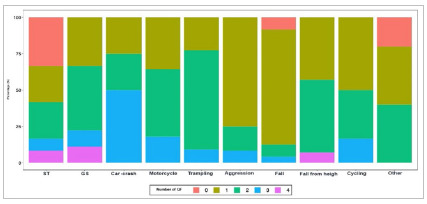
Frequency of trauma mechanism versus Number of QF compromise (p<0.001,Chi-square) at traumatized in-patients; ST - Stabbing; GS - Gunshot.

The number of compromised QFs was directly related to the morbidity of these patients, with a greater number of complications (p=0.075) and longer hospital stay (p=0.028), although not directly related to mortality (p=0.564).

## DISCUSSION

The implementation of the Trauma Registry in the Emergency Service of the Irmandade da Santa Casa de Misericórdia de São Paulo, through the acquisition of automated data by the REDCap platform, demonstrated that this tool is adequate and ideal for data collection, allowing the confidentiality of trauma analysis data, assured by the platform itself, and, from an operational point of view, the analysis of complex and numerous data in an objective way.

Its implementation in a pilot period underwent adaptations that allowed the identification of points of failure in data collection and storage, but which ultimately culminated in the methods set out here to consolidate the institution’s Trauma Registry. The lack of solid data in the medical records on the Respiratory Rate of patients suffering from mild trauma admitted on spontaneous demand can be cited, which made it impossible to classify their severity using any physiological index, such as the ISS. Therefore, a specific Quality Filter was created for this parameter and educational rounds were carried out to raise awareness of the care teams.

When analyzing the population sample from the Trauma Registry, we found it to be representative when compared with the main national and international trauma registries^2^ ^,^ ^3^ ^,^ ^31^ ^,^ ^32^: the majority of trauma victims are young men, with a large number of car accidents and predominance of traumas with lower kinetic energy, when compared with severe traumas; there was a relatively low incidence of penetrating injuries, even considering the Brazilian reality as a high incidence of interpersonal violence. This fact is relevant, not only for validating the data obtained, but also for understanding the dynamics of the service under study, since despite being a reference center for trauma patients, with attributes for classification as “Level 1 Trauma Center”, it is a philanthropic institution with an “open door” Emergency Service, that is, with care based on spontaneous popular demand and a non-referenced pre-hospital care service, allowing care for severe and mild traumas, generating a non-biased sample of the Trauma Disease. Furthermore, the period studied includes one of the major peaks in contagion of the SARS-Cov2 (COVID-19) Pandemic, which greatly reduced the incidence, the need for emergency intervention, and hospital admission due to serious trauma.

The use of Quality Filters as points of care in the trauma patient care chain had already been consolidated in previous research^19^ ^,^ ^27^ ^,^ ^35^ ^,^ ^37^ ^,^ ^43^ ^,^ ^44^ with smaller populations. However, its validation in the selected sample, through representativeness and segmental analysis, endorses the use of these parameters as a mechanism for detecting service failures in trauma victims in the Brazilian reality.

In the assessment of the impairment of Quality Filters in patients’ morbidity and mortality, we opted to analyze only hospitalized patients to avoid sampling and performance biases, since those patients whose hospitalization is brief are generally victims of mild trauma and susceptible to fewer complications and to more successful final treatment.

When analyzing the Quality Filters individually, we observed that the presence of an unnoticed lesion in the first 24 hours (FQ1), Subdural Hematoma drainage time >4h (FQ2), non-activation of the Massive Transfusion Protocol (FQ6), Absence of Respiratory Frequency record at admission (FQ7), and Change in vital signs without analysis of arterial blood gases and lactate (FQ8) did not demonstrate significant failures. This result reflects the implementation of specific trauma care protocols for more than 10 years in the ISCMSP Emergency Service and the study of the quality of trauma care as the main objective^34^.

Chest re-drainage (FQ3) was not related to the severity of the trauma, the severity of the thoracic injury, or morbidity and mortality outcomes. This result shows that the compromise of this point of care is probably related to pleuropulmonary complications, as illustrated in the study by Nascimento IKD in 2022^39^, inherent to being performed in a trauma room and in an emergency scenario.

The fact that patients with open fractures went to the OR in more than 6 hours (FQ4) was directly related to the incidence of complications during hospitalization, a fact that did not change the length of stay or patient mortality. This data correlates with the trauma mechanism - car accidents. Such patients present multiple injuries in multiple body segments, despite the severity observed in the extremities. In the context studied, the process of investigating other potentially life-threatening injuries ends up delaying the surgical approach to open fractures, as well as impacting the quality of care in other ways, corroborated by the number of Quality Filters compromised in this patient profile. This Quality Filter proved to be valid, from a statistical point of view, as a point of analysis of the quality of the care provider chain.

Referral of patients in shock to Exploratory Laparotomy within the first hour (FQ5) was compromised in two patients. When analyzing each of the cases, one was responsive to fluid resuscitation, being operated on for peritonitis due to small ischemia secondary to vascular injury of the mesentery; in the second, we noticed a transient response to initial resuscitation which, associated with the involvement of multiple segments, led to additional investigation, delaying definitive management. Therefore, considering this failure, the existence of a hybrid room, or resuscitation in Operating Room^40^, could reduce the delay in the chain of care for this subgroup of patients and, possibly, reduce associated complications.

Although the Filter related to the CT Report delivered in >6h (FQ10) does not have any relationship with outcome or length of hospital stay, it is worth considering that the analysis of this parameter in a population of patients with clinical indication for hospital admission, whether due to their severity or to the demand for surgical treatment, constitutes a selection bias in the analysis of this specific treatment, since the duration of hospitalization will be related to the injuries present and will be little influenced by the delay in preparing the CT report. Future analysis of this Filter in the subpopulation of non-hospitalized patients may provide more information about its impact on hospital stay.

The prevalence and relevance of trauma as a factor in mortality and loss of productive years in young patients makes measures to improve outcomes fundamental points of attention for these patients. A mature Trauma Registry is the cornerstone of any measure aimed at improving care. Despite possible biases or flaws, as a set of measures or analyzed separately, through the data presented, Quality Filters can be attributed the ability to identify flaws and promote improvements in trauma care in services with well-established Quality Program and Trauma Registry.

## CONCLUSION

The use of the REDCap Platform is suitable for implementing Trauma Records in smaller services, as well as playing a fundamental role in scientific production in services dedicated to trauma care. Quality Filters also demonstrated relevance in the analysis of possible improvements in the care of trauma victims, especially those of greater severity, as stratified by ISS/NISS. The implementation of Quality Filters through Trauma Records is a fundamental tool in the quality of care for traumatized patients.
